# Predicting persistent pain after total knee arthroplasty using different machine learning algorithms

**DOI:** 10.2340/17453674.2026.45965

**Published:** 2026-06-22

**Authors:** Anni RAJAMÄKI, Aleksi REITO, Mari KARSIKAS, Mika NIEMELÄINEN, Antti ESKELINEN

**Affiliations:** Coxa Hospital for Joint Replacement and Faculty of Medicine and Health Technology, Tampere University, Tampere, Finland

## Abstract

**Background and purpose:**

After total knee arthroplasty (TKA), 10–20% of patients remain unsatisfied. Well-performing clinical prediction models can provide individualized risk estimates and stratification in terms of poor outcomes, resulting in unnecessary surgeries being avoided and patients being counseled preoperatively. We aimed to create a precise, well-performing prediction model for clinical application using different machine learning algorithms to predict those patients who will have residual pain, a low total Oxford Knee Score (OKS) and the patient group who do not achieve minimally clinical important difference (MCID) in OKS 1 year after TKA.

**Methods:**

We conducted a retrospective cohort study based on patients who had undergone primary TKA at our institution combined with 751 patient-related variables. The multivariable models used were based on the results of univariate analysis. We used the machine learning method Extreme Gradient Boosting (XGBoost). The discrimination capability of the models was measured with the area under the curve (AUC).

**Results:**

11,755 patients were included in this study. There were 850 (7.2%) patients who experienced persistent pain 1 year after TKA. The AUC was 0.67. For the secondary outcomes, the AUC values were similar. The most important variables in the model were lower preoperative OKS, younger age, valgus malalignment, lower preoperative pain OKS, use of mild opioid, neuropathic pain medicine and thyroxine, and higher body mass index.

**Conclusion:**

The prediction models achieved poor AUCs. It seems clear that the prediction of pain and functional outcome after TKA is difficult, even with a large patient cohort combined with 751 patient-related variables and sophisticated machine-learning algorithms.

Total knee arthroplasty (TKA) is a highly successful and cost-efficient treatment for patients who have end-stage knee osteoarthrosis (OA) Nevertheless, approximately 10–20% of patients remain unsatisfied and around 25% will have chronic pain after TKA surgery [1-3]. Unsatisfied patients report residual symptoms such as pain, stiffness, and compromised performance of the knee [3,4].

Recently, interest has shifted towards methods using machine learning and artificial intelligence to develop prediction models for different outcomes in medical research. Well-performing clinical prediction models can provide individualized risk estimates and stratification in terms of poor outcomes, resulting in unnecessary surgeries being avoided and patients being counseled preoperatively. The identification of potentially modifiable risk factors for poor outcomes would also be useful step.

Machine learning methods have previously been used to predict different outcomes of TKA. Prior attempts have been made to create prediction models for unsatisfied patients who experience postoperative pain after TKA with moderate prediction capability (area under the curve [AUC] or c-statistics 0.7–0.8) [5–12]. The outcomes and predictors used in these earlier studies have varied and the size of the patient cohorts has been limited (n = 400–1,500). The predictors in these studies have usually been basic demographics such as age, sex, ASA class, body mass index (BMI), socioeconomic status with some additional information (including information on opioid usage), history of different medical comorbidities, and patient-reported outcome measures (PROMs).

We aimed to create a precise, well-performing prediction model for clinical application using different machine learning algorithms to primarily predict those patients who will have residual pain, and secondarily to predict who will have a low total Oxford Knee Score (OKS) and the patient group who do not achieve minimally clinical important difference (MCID) in OKS 1 year after TKA.

## Methods

### Study design and participants

**Figure 1:**
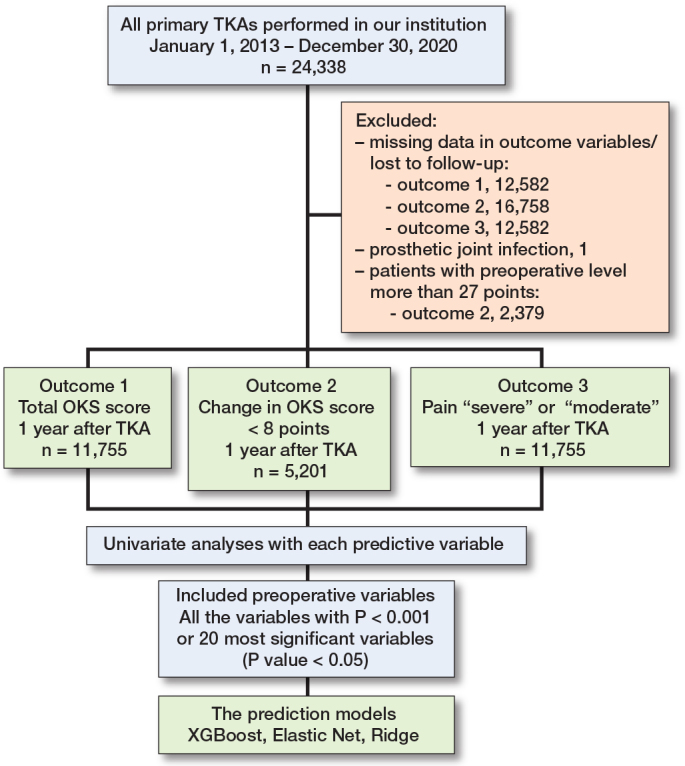
Flowchart of the study.

This retrospective cohort study was based on register data from a high-volume academic joint replacement center. The data included all primary TKAs performed between January 1, 2013, and December 30, 2020. At least 1 year of follow-up was required. Only patients with primary osteoarthritis were included (Figure 1). The TRIPOD guideline was used for reporting.

### Dataset

The dataset was gathered from the total joint arthroplasty data registry at our institution. The registry contains all the prospectively collected preoperative, intraoperative, and postoperative information on each surgery from the electronic health record systems (Pegasos [CGI, Canada], Uranus [CGI, Canada], Tekoset [Coxa, Finland], and Centricity Opera [GE Healthcare, USA]). The registry constantly receives updates from the Digital and Population Data Services Agency on the possible deaths and emigrations of patients. There were a total of 751 preoperative variables. Of these variables, 469 were different medications classified by the Anatomical Therapeutic Chemical (ATC) Classification (WHO) system. In addition, there were also 131 medical comorbidities and allergies as well as 30 different preoperative blood tests. The remaining variables were the basic demographics of age, sex, American Society of Anesthesiologists Physical Status (ASA) class, and BMI. Information on previous knee injuries, smoking, operating surgeon (personal code for each surgeon, information on teaching surgery), wound closure technique, information on preoperative clinical examination of the knee joint (stability, range of movement, degree of malalignment, pulsation of arteria dorsalis pedis and tibial posterior arteries, patellar status) was obtained from the Tekoset system. The anesthesia management system contained information on the anesthesia used.

### Outcome

The Oxford Knee Score (OKS) was used as the primary outcome measure. The OKS is a patient-reported outcome measure (PROM) for patients who undergo TKA. It consists of 12 questions, each having 5-step ordinal answers (0–4). The questions are focused on assessing the pain and function of the TKA [13,14]. The OKS questionnaires were collected preoperatively and postoperatively after 2–3 months and at 1 year. We used the postoperative scores collected at 1 year after surgery in this study. The first question on the OKS deals with the total pain experienced 1 year after surgery. This consists of 5 ordinal answers that rate the pain as severe, moderate, mild, very mild, or no pain. The primary outcome was to predict the patient group who reported residual pain to be “moderate” or “severe”.

In the secondary outcome, poor postoperative OKS was considered to be < 25 out of a total 48 points. The threshold value was based on clinical experience of poor OKS scores as well as the earlier literature on what is considered a treatment failure (TF) score [15]. The MCID for OKS is 9 points at group level analysis and 7 points when comparing the change at the individual level [16]. In the other secondary outcome, if the change from preoperative level was under 9 points, the patient was considered to have not received enough benefit from the surgery. Also, if the patient had lower OKS postoperatively than the preoperative level, the patient was also placed into this group. This secondary outcome—to predict the group who had MCID under 9 points—was predicted from the population who had preoperative OKS < 28 points. Therefore, only patients with room for improvement without the risk of ceiling effect were included in this analysis [14,17–19].

### Predictors

Predictors for the model were selected based on univariate analysis results. Univariate analysis was performed with all the available preoperative data and all the outcome variables. The variables that had P < 0.001 or at least 20 of the most significant variables (still P < 0.05) were selected for the multivariable model.

### Missing data

Missing covariate data was handled as a separate variable “no value.” Patients with any missing data on outcome variables were excluded. The missing data was assumed to be missing completely at random [20]. The numbers are presented in the flowchart (see Figure 1).

### Data analyses

The Extreme Gradient Boosting (XGBoost) method was used for the prediction model. XGBoost is an advanced ensemble machine learning algorithm that employs a gradient boosting framework on decision trees [21]. It is recognized for its efficiency, scalability, and ability to handle sparse data, making it particularly suitable for the electronic health record dataset used in this study [22]. To optimize the performance of the XGBoost model, hyperparameters, such as the learning rate, number of trees, and tree depth, were tuned using a grid search approach. To improve interpretability of the XGBoost prediction model, feature contributions were evaluated using Shapley additive explanations (SHAP). SHAP values quantify the contribution of each feature to the model prediction for individual observations. We complemented the use of the XGBoost algorithm with Ridge Regression and Elastic Net as reference methodologies. Ridge Regression addresses multicollinearity in datasets with highly correlated predictors by introducing a penalty term [23]. Elastic Net, merging the features of Ridge and Lasso Regression, applies so called L1 and L2 penalties to achieve sparse model solutions and robust feature selection, which is invaluable in handling complex medical datasets with numerous predictors [24].

To ensure the effectiveness and generalizability of the predictive models developed in our study, the dataset was divided into 2 subsets by a random selection. The training set, comprising 70% of the data, was employed for model training. The remaining 30% of the data formed the testing set, which was used exclusively for model evaluation. The reported AUC values are based on the testing data results only.

To evaluate the performance of the predictive models, we employed a couple of key metrics, the Area Under the Receiver Operating Characteristic (AUC-ROC) curve and calibration curves, alongside analyses of relative significance.

All the analyses were performed with R Statistical software (R Foundation for Statistical Computing, Vienna, Austria) using the packages ggplot2, doParallel, gbm, pROC, glmnet, doMC, classifierplots, xgboost, and plotROC.

### Ethics, data sharing plan, funding, and disclosures

In accordance with Finnish legislation on clinical research, no ethical committee approval or informed written consent was required because of the retrospective register-based study design. Permission to conduct this study was granted by the institutional review board at our institution.

This study was funded by the competitive research funds of Pirkanmaa Hospital District, Tampere, Finland (representing governmental funding), the Finnish Research Foundation for Orthopaedics and Traumatology, the Finnish Medical Foundation, and the Finnish Arthroplasty Association. The funding sources played no role at any stage of the study.

Complete disclosure of interest forms according to ICMJE are available on the article page, doi: 10.2340/17453674.2026.45965

## Results

We included 11,755 primary TKAs in the final analysis. In this cohort, there were 850 (7.2%) patients who reported either “moderate” or “severe” knee pain 1 year after TKA surgery. The median postoperative OKS was 43 points (interquartile range [IQR] 38–46). In the analysis of predicting patient group with OKS change less than MCID, there were 5,201 TKAs, and the median change the patients obtained in OKS was 23 points (IQR 18–27), clearly exceeding the MCID of 9 points (Table 1, Appendix 1). In addition, 85% of the available data provided information on anesthesia, where 1.5% comprised general anesthesia, 85.3% a combination of spinal and epidural anesthesia, 8.6% spinal, and 4.6% spinal with catheter.

**Table 1:** Characteristics of the patient cohort (N = 11,755)

Variable	Values	Missing data, n
Age, mean (SD)	67 (9.4)	0
Female sex, %	63	0
Body mass index, mean (SD)	30.5 (5.5)	171
ASA classification, n		31
1	675	
2	5,183	
3	4,962	
4	930	
5	0	
Total OKS, median (IQR)		
1 year postoperatively	43 (38–46)	0
preoperative	21 (16–24)	0
Achievement in OKS, mean	20 (14–26)	0

ASA = American Society of Anesthesiologists Physical Status;

IQR = interquartile range; OKS = Oxford Knee Score;

SD = standard deviation.

### Residual knee pain at 1 year after TKA surgery

**Figure 2:**
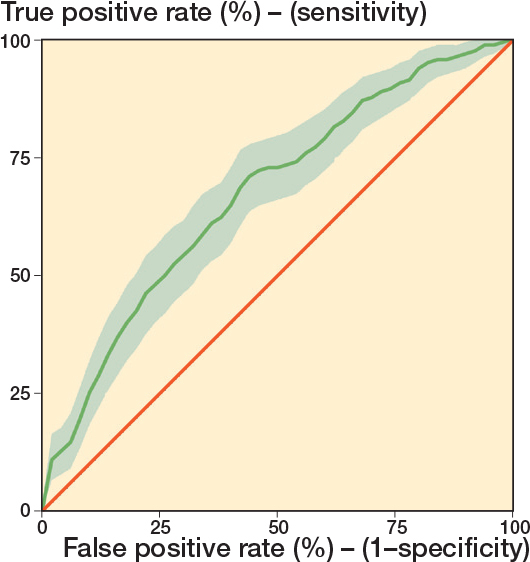
Severe or moderate pain remains: ROC curve. AUC 0.67 (95% confidence interval 0.63–0.72).

**Figure 3:**
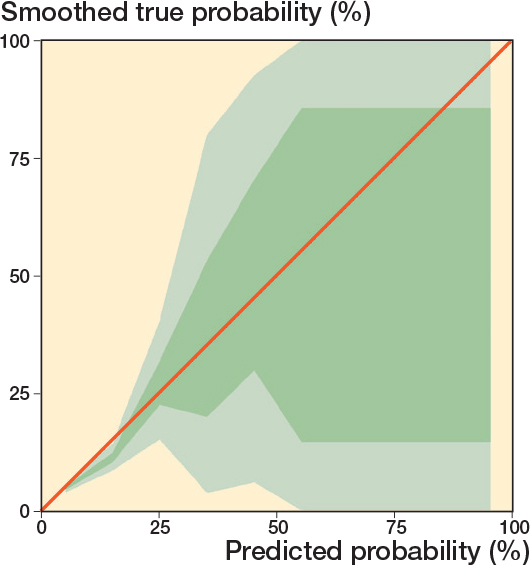
Severe or moderate pain remains: calibration graph.

There were 3,900 patients in the training set, where 281 (7.2%) reported pain as “moderate” or “severe.” The testing set consisted of 2,574 patients, where 162 (6.3%) had “moderate” or “severe” pain. The AUC was 0.67 (95% confidence interval [CI] 0.63–0.72) (Table 2, Figure 2). The calibration graph shows that the model is overestimating the prediction capability (Figure 3).

**Table 2:** Area under the curve (AUC) values of the prediction models

Outcome	AUC (CI)
Total OKS 1 year postoperatively (n = 11,755)	
XGBoost	0.68 (0.63–0.73)
Ridge	0.66 (0.61–0.72)
ElasticNet	0.67 (0.61–0.72)
The change in OKS from preoperative level (n = 5,201)	
XGBoost	0.68 (0.63–0.73)
Ridge	0.66 (0.60–0.71)
ElasticNet	0.64 (0.58–0.70)
The presence of “moderate” or “severe” pain 1 year postoperatively (n = 11,755)	
XGBoost	0.67 (0.63–0.72)
Ridge	0.67 (0.63–0.71)
ElasticNet	0.65 (0.61–0.69)

OKS = Oxford Knee Score.

**Figure 4:**
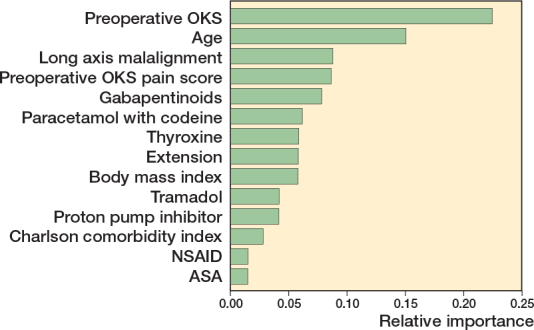
Relative importance between variables in the prediction of severe or moderate pain remains.

The most important variables predicting a negative outcome were lower preoperative OKS, younger age, valgus malalignment, lower preoperative pain OKS, use of mild opioid, neuropathic pain medicine and thyroxine, and higher BMI (Figure 4, Table 3).

**Table 3:** SHAP (Shapley additive explanations) values

Variable	SHAP value
Long axis malalignment	0.21
Preoperative OKS	0.19
Age	0.18
OKS pain score preoperatively	0.08
Charlson comorbidity index	0.07
PPI	0.07
Paracetamol with codeine	0.04
ASA score	0.04
Thyroxine	0.03
Extension	0.02
Tramadol	0.02
Body mass index	0.02
NSAID	0.02
Gabapentinoids	0.006

For abbreviations, see Table 1.

### Prediction of low total OKS score

**Figure 5:**
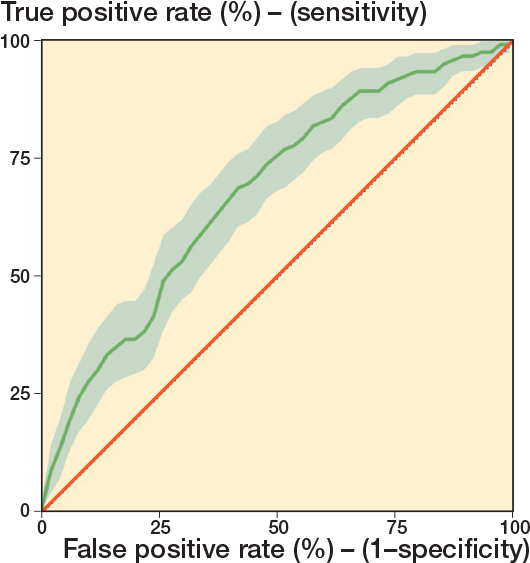
Prediction of total OKS: ROC curve. AUC 0.68 (95% confidence interval 0.63–0.73).

**Figure 6:**
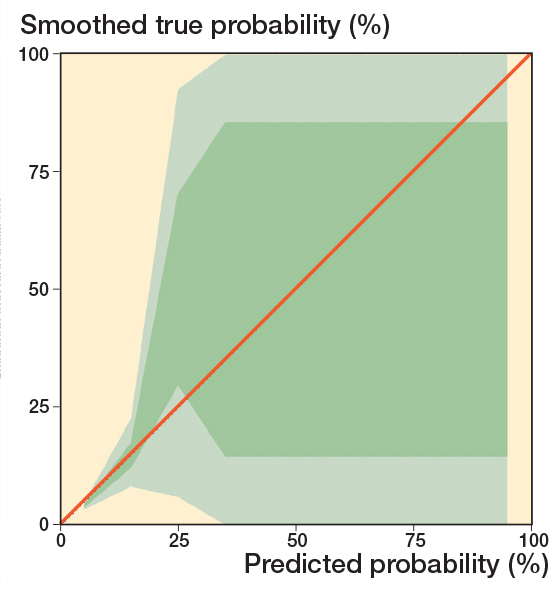
Prediction of total OKS: calibration graph.

**Figure 7:**
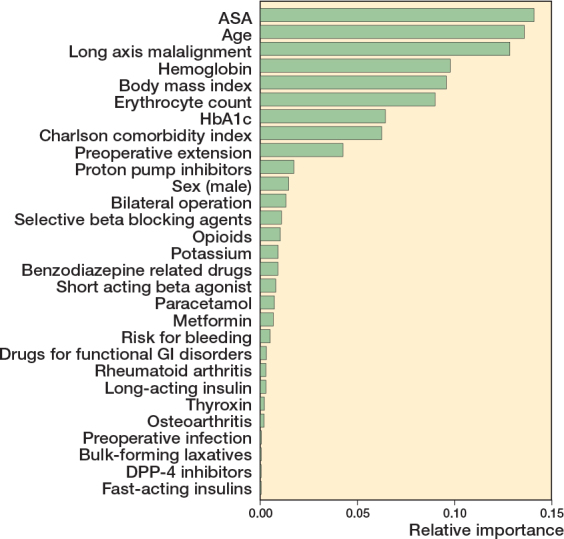
Relative importance between variables in the prediction of total OKS.

In this secondary outcome, the training set consisted of 9,000 (77%) participants of whom 392 (4.3%) had a 1-year postoperative OKS of < 25 points (see Table 2). The testing set had 2,755 participants and 119 of these had an OKS of < 25 points (4.3%). The AUC was 0.67 (CI 0.62–0.72) with the testing set (Table 2, Figure 5). A calibration graph (Figure 6) shows that the model is underestimating the prediction capability in the testing set. The most important variables predicting a negative outcome were higher ASA, wider malalignment in preoperative long-axis plain radiographs, younger age, presence of anemia, and higher whole blood HbA1c levels (Figure 7, Appendix 2).

### Prediction of OKS change less than MCID

**Figure 8:**
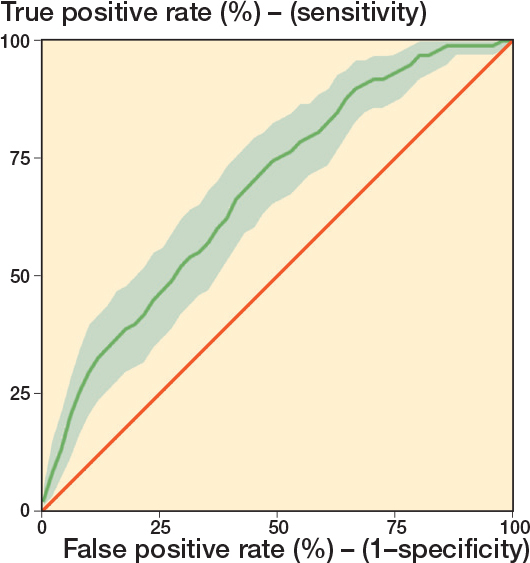
Change in OKS from the preoperative level: ROC curve. AUC 0.68 (95% confidence interval 0.63–0.73).

**Figure 9:**
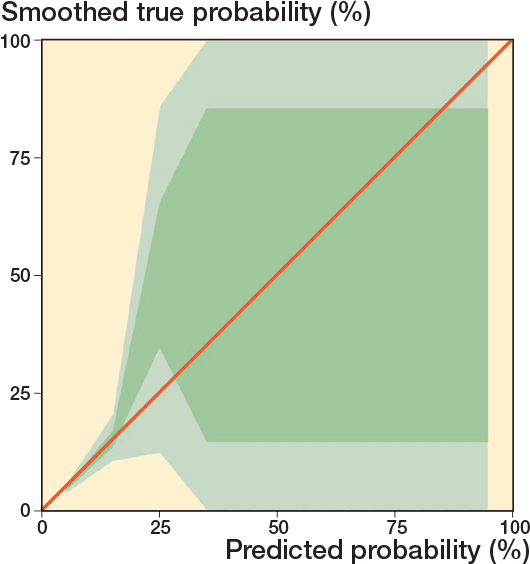
Change in OKS from the preoperative level: calibration graph.

**Figure 10:**
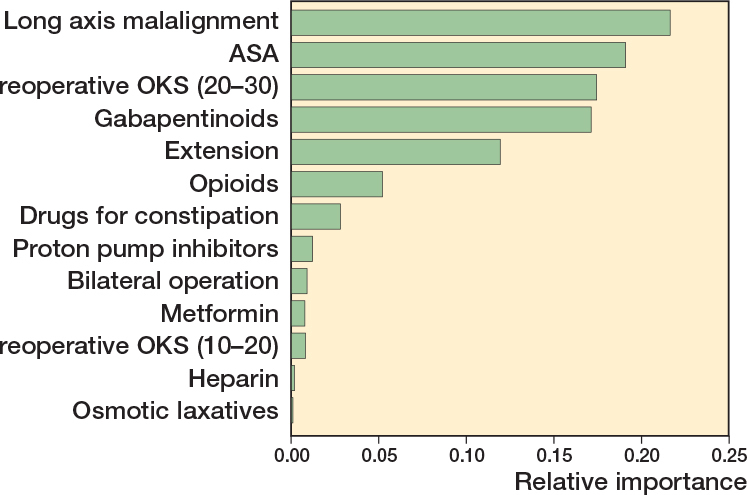
Relative importance between variables in the prediction of the change in OKS from preoperative level.

This secondary outcome had a training set 3,500 (67%), where 225 (6.4%) had a difference of ≤ 8 points after surgery. The testing set had 97 poor outcomes (5.7%) out of 1,701 participants. The AUC was 0.68 (CI 0.63–0.73) with the testing set (see Table 2, Figure 8). In the calibration graph, the predicted risk is underestimated (Figure 9). The most important variables predicting a negative outcome were higher ASA, malalignment in the preoperative long-axis plain radiographs, low preoperative OKS, and the use of neuropathic and opioid pain medication (Figure 10, Appendix 3).

### Sensitivity analysis

We also conducted the same analyses using the Ridge and Elastic Net methods. The AUC were similar and did not process any better (see Table 2).

## Discussion

This is the first study to develop ML models aimed at predicting TKA outcomes using the OKS questionnaire.

The aim of this study was to develop a clinically useful prediction model that could aid surgeons in patient selection for primary TKA, and in particular to help them better identify those patients who are not likely to benefit from such surgery. We found poor AUCs for all the tested models (0.67–0.68) in our study. This happened even though we had a large patient cohort from 1 hospital, a dataset with a large amount of different clinical, laboratory, and even imaging information, and which used sophisticated statistical methods. The AUCs achieved with these methods were similar, and using the most sophisticated method did not improve the results. Indeed, the most important variables—ASA, preoperative malalignment, low preoperative OKS, use of neuropathic or opioid pain medication—were similar in all models.

In the systematic review by Hinterwimmer et al. (2022), different outcomes of TKAs predicted by machine learning methods were investigated and achieved an AUC median of 0.76. The studied outcomes were complications, costs, functional outcome, revision, satisfaction, surgical technique, and biomedical properties [25]. In a study by Harris et al. (2021), prediction models for KOOS and its subscore that deals with pain were created. The predictors included in that study were age, sex, ethnicity, marital status, level of education, and employment status. The c-statistics were 0.71–0.76 [5]. Klemt et al. studied different PROMs, such as HOOS-PS, KOOS-PS, Physical Function SF10A, PROMIS SF Physical, and PROMIS SF Mental, using 4 different machine learning methods and found an AUC of > 0.83. The included predictors were ASA, BMI, age, length of hospital stay, insurance status, and ethnicity [10]. Other studies have also created prediction models for patient satisfaction using different PROMs. In those studies, the c-statistics or AUC have implicated moderate prediction capability, with an AUC of 0.7–0.8 or c-statistics of 0.7–0.8. In all previous studies, both the size of the patient cohort and the available predictors have been remarkably smaller than in our study [6,11,12]. We are unaware of any previous ML prediction model studies that have used the OKS as the primary outcome measure.

Zhang et al. investigated patient satisfaction prediction models after total hip arthroplasty (THA) using 4 different methods, with LASSO managing the best with an AUC of 0.76, followed by XGBoost with an AUC of similar to our 0.66 [26]. In our study, we had almost 12,000 patients with 751 preoperative variables of patient-related information such as medication, medical comorbidities, and laboratory tests. However, the prediction capacity remained low in all models. In our study, we had clinical variables available similar to those in the earlier literature. However, even after the addition of medication, medical comorbidities, and laboratory tests, we were still unable to achieve results that were superior to those of the earlier studies. First, using a larger dataset may introduce more heterogeneity among patients, which can reduce the apparent predictive performance of the models. In addition, larger datasets allow for better adjustment for potential confounders, which may attenuate overly optimistic results seen in smaller or less representative cohorts. While these factors may contribute to poorer model performance, they may also result in more realistic estimates that better reflect real-world clinical practice.

Although interest is now focused on prediction models that are based on machine learning and artificial intelligence methods, there is still a lack of evidence that these methods have the capability of reliably predicting patient dissatisfaction or residual pain after TKA. One reason for this could be the very subjective elements of experienced pain and satisfaction, which makes it difficult to decide on threshold levels for PROMs such as the OKS. The preoperative OKS was an important predictive variable showing that low OKS may be due to reasons other than knee OA, and that the time-dependent and varying nature of knee OA may also have an effect. These factors can also affect the results of the prediction model. Therefore, a lower AUC could be accepted when building a theoretical prediction model. When the aim is to build a prediction model for clinical decision-making, however, high AUCs should still be the goal.

### Strengths

The strength of our study is our large single-center data pool, where information is gathered in a structured form, providing us with good facilities for building prediction models using very specific patient-related information. However, our high surgical volume may also affect generalizability of our findings as the patient care protocols and clinical practice may differ in lower volume hospitals. We also compared the different machine learning methods and used model validation. However, the dichotomous nature of our outcome variables is a limitation that must be considered. For secondary outcomes, we had to predict an “unsatisfactory outcome” that was based on the previous literature instead of keeping them linear because the prediction model is more capable of working with logistic variables than linear ones.

### Limitations

It should also be noted that there are some limitations with the OKS. First, there is a ceiling effect in the OKS that was managed by excluding those patients with preoperative scores over 28 points. Therefore, there is a room for improvement without the risk of a ceiling effect. Second, as the OKS is a PROM, it is a subjective measurement. Patients have different kinds of needs or expectations for pain and function after TKA, which may have affected the score. In a study by Vega et al., it was reported that socioeconomic factors (younger age, obesity, non-White race, female sex, current or recent smoking, non-commercial insurance, and increased CCI) were associated with worse results of the KOOS questionnaire. It was also debated whether people from a lower socioeconomic class tend to seek a doctor’s help later [27]. Interestingly, patients from higher socioeconomic classes are often more interested in answering PROMs, which can also affect selection bias [28]. Also, in this study, there were over 12,000 patients excluded who did not have the information on OKS available, so, as mentioned earlier, that might affect the selection bias.

### Conclusion

We only succeeded in creating prediction models with rather low AUCs. Patient selection for TKA is complex, and, despite the knowledge and tools that we have available nowadays, it is still hard to predict those patients who are likely to experience pain after primary TKA and not benefit from the procedure.

### Supplementary data

Appendices 1–3 are available as Supplementary data on the article homepage, doi: 10.2340/17453674.2026.45965

## Supplementary Material



## Notes

ARa: formal analysis, visualization, writing—original draft. ARe: conceptualization, methodology, writing—review and editing. MK: data curation, methodology, formal analysis, visualization, writing—review and editing. MN: writing—review and editing. AE: conceptualization, supervision, writing—review and editing

The authors would like to thank Peter Heath, MA, for linguistic help.

Handling co-editors: Ivan Hvid and Robin Christensen

*Acta* thanks Leif Ryd and B Willem Schreurs for help with peer review of this manuscript.
