# Supplementary material for: Predicting persistent pain after total knee arthroplasty using different machine learning algorithms
Source: Acta Orthop. 2026 Jun 22;97:423–9. doi: 10.2340/17453674.2026.45965 (PMC13285364; doi:10.2340/17453674.2026.45965)
Supplement: Supplementary file 1 [file ActaO-97-45965-s1.pdf]

Supplementary data

Appendix 1. Covariate descriptives

Outcome 1. Clinical failure, defined as moderate or severe knee pain 1 year postoperatively, based on item 1 of the OKS

Variable	Count (yes / no)	Missing values
Proton pump inhibitors	3,532 / 8,213	12
Thyroxin	1,479 / 10,266	12
NSAID	3,278 / 8,467	12
Paracetamol with codeine	893 / 10,852	12
Tramadol	482 / 11,263	12
Gabapentinoids	393 / 11,352	12

Outcome 2. Total OKS 1 year postoperatively

Variable	Median (IQR) / Count (yes / no)	Missing values
Long axis malalignment °, median (IQR)	4 (0–7)	0
Hemoglobin, g/L, median (IQR)	139 (131–147)	159
Erythrocyte count, 10^6 cells/µL, median (IQR)	4.6 (4.3–4.9)	159
HbA1c, mmoL/moL, median (IQR)	38 (35–41)	655
Preoperative extension, median (IQR)	0 (0–5)	0
Risk for bleeding preoperatively assessed	1,628 / 9,992	135
Bilateral operation	1,689 / 10,066	0
Proton pump inhibitor medication	3,529 / 8,214	12
Selective beta blocking agents	2,610 / 9,133	12
Opioids	1,927 / 9,816	12
Potassium	182 / 11,561	12
Benzodiazepine related drugs	689 / 11,054	12
Short acting beta agonist	757 / 10,986	12
Paracetamol	5,765 / 5,978	12
Metformin	1,194 / 10,549	12
Drugs for functional GI disorders	265 / 11,478	12
Long-acting insulin	292 / 11,451	12
Thyroxin	1,479 / 10,264	12
Bulk-forming laxatives	199 / 11,544	12
DPP-4 inhibitors	346 / 11,397	12
Fast-acting insulins	133 / 11,610	12

DPP-4 inhibitors = dipeptidylpeptidas 4 inhibitor

Outcome 3. The difference achieved in the OKS 1 year after operation compared with the preoperative score

**Variable**	**Median (IQR) / Count (yes / no)**	**Missing values**
Long axis malalignment °, median (IQR)	4 (0.0–7.0)	0
Extension °, median (IQR)	0 (0.0–5.0)	0
Bilateral operation	721 / 4,477	0
Drugs for constipation	439 / 4,759	3
Heparin	326 / 4,872	3
Opioids	458 / 4,740	3
Gabapentinoids	200 / 4,998	3
Metformin	541 / 4,657	3
Osmotic laxatives	242 / 4,956	3
Proton pump inhibitors	1,406 / 3,792	3
Preoperative OKS		0
0–10	421	
10–20	2,604	
20–30	2,176	

Appendix 2. SHAP-values for predicting total OKS 1 year after TKA

Variable	SHAP-value
ASA score	0.16
Long axis malalignment	0.12
Body mass index	0.12
Age	0.09
Erythrocyte count	0.09
Hemoglobin	0.08
Extension	0.08
Charlson comorbidity index	0.06
HbA1c	0.06
Opioids	0.04
Proton pump inhibitors	0.03
Sex male	0.03
Bilateral operation	0.03
Benzodiazepine related drugs	0.02
Metformin	0.01
Long-acting beta agonist	0.01
Potassium	0.01
Short acting beta agonist	0.01
Thyroxin	0.01
Risk of bleeding	0.01
Paracetamol	0.004
Medication for gastrointestinal disorders	0.004
Long-acting insulin	0.004

Appendix 3. SHAP-values for predicting the change in total OKS from preoperative level

Variable	SHAP-value
ASA score	0.29
Preoperative OKS (20–30)	0.18
Extension	0.15
Long axis malalignment	0.13
Opioids	0.06
Gabapentinoids	0.03
Metformin	0.02
PPI	0.02
Constipation drugs	0.02
Heparin	0.01
Preoperative OKS (10–20)	0.01
Laxatives	0.004
Bilateral operation	0.002
